# Joint Optimization for Task Offloading in Edge Computing: An Evolutionary Game Approach

**DOI:** 10.3390/s19030740

**Published:** 2019-02-12

**Authors:** Chongwu Dong, Wushao Wen

**Affiliations:** School of Data and Computer Science, Sun Yat-Sen University, Guangzhou 510006, China; dongchw@mail2.sysu.edu.cn

**Keywords:** task offloading, mobile edge computing, evolutionary game theory

## Abstract

The mobile edge computing (MEC) paradigm provides a promising solution to solve the resource-insufficiency problem in mobile terminals by offloading computation-intensive and delay-sensitive tasks to nearby edge nodes. However, limited computation resources in edge nodes may not be sufficient to serve excessive offloading tasks exceeding the computation capacities of edge nodes. Therefore, multiple edge clouds with a complementary central cloud coordinated to serve users is the efficient architecture to satisfy users’ Quality-of-Service (QoS) requirements while trying to minimize some network service providers’ cost. We study a dynamic, decentralized resource-allocation strategy based on evolutionary game theory to deal with task offloading to multiple heterogeneous edge nodes and central clouds among multi-users. In our strategy, the resource competition among multi-users is modeled by the process of replicator dynamics. During the process, our strategy can achieve one evolutionary equilibrium, meeting users’ QoS requirements under resource constraints of edge nodes. The stability and fairness of this strategy is also proved by mathematical analysis. Illustrative studies show the effectiveness of our proposed strategy, outperforming other alternative methods.

## 1. Introduction

Mobile applications with immersive experience are becoming popular and will be killer applications in 5G networks. According to the work [1], the global market in augmented reality (AR) and virtual reality (VR) will develop quickly in the future world. Other applications, such as online games and mobile health care, also developed rapidly in the past few years. Nowadays, diverse commercial devices, such as Google’s Glass, Facebook’s Oculus, Samsung’s Gear VR, and HTC’s Vive, are now available to support applications with immersive experience in real time.

However, there are still some problems to enable these new types of applications to support user mobility. For example, a general VR system, such as HTC’s Vive, captures interactive tracking from lots of sensors and uploads the tracking into dedicated servers for further processing. Then, the rendered high-resolution 360-degree video would be streamed back to the headset by high-speed HDMI cables. Although the wired cables reduce the delay for transferring huge amount of data, they limit a player’s mobility and affect user experience seriously. To enhance users’ mobility, an untethered solution, which utilizes high-performance mobile devices, is proposed in the industrial and academia. However, limited battery life and constrained computation capacity in a mobile device make this solution non-ideal in practice as a resource-hungery application may include different types of computation-intensive tasks, such as frame preprocessing, object tracking, and annotation rendering. According to the measurement in Ref. [2], a high-performance mobile phone can only support up to 2 h of Pokemon Go, a famous mobile AR game. Depending on the local computation capacity of mobile terminals, this is far from enough to serve these heavy applications well.

**Figure 1:**
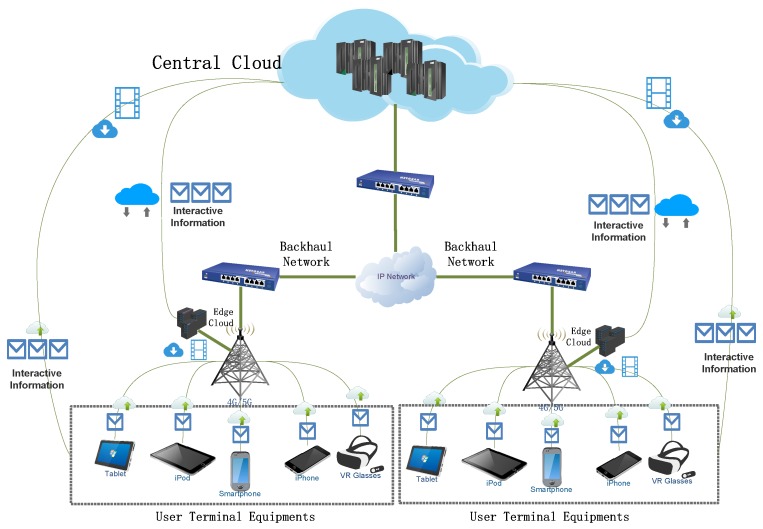
An overview of a mobile edge computing (MEC) system.

To tackle the problem of limited resources in a mobile device, offloading tasks to clouds is an efficient way to augment the computation capacity of a mobile device. However, due to the geographically diverse distance from different mobile users to cloud data centers, it is difficult to guarantee similar proper response time for all users. Actually, traditional cloud infrastructure may not satisfy the stringent delay requirements for such mobile applications. To mitigate the problem of latency constraint for delay-sensitive applications, a new cloud paradigm, named mobile edge computing (MEC), have been proposed [3]. In the field of academia, MEC is also similar to fog computing [4], cloudlet [5], small cell cloud [6], and Follow me Cloud [7]. In a MEC cloud platform, a number of small scale and dedicated servers are deployed at the network edge close to mobile users. Users can run an immersive application smoothly in their mobile handset by offloading computation-intensive tasks to edge clouds in close proximity to them. One or more large scale cloud data centers, called central clouds, to cooperate and synchronize with edge nodes, are also included in a MEC cloud platform. Figure 1 shows an example of a MEC cloud platform.

In the MEC cloud platform, there still exist some practical challenges for mobile devices to perform task offloading successfully. First, resources in each individual edge cloud may be insufficient to serve growing number of mobile devices. One edge cloud cannot undertake all offloaded tasks that exceeds its computation capacity. Coordinating multiple edge clouds together to serve multi-users from different regions should be taken into consideration. A central cloud shall also be involved in task processing for mobile users. Therefore, a task may be offloaded to different target processing places including a mobile user’s serving MEC node, a nearby MEC node, or a node at a center cloud. Second, different usage cost may be incurred when tasks are offloaded to different target processing places. In terms of minimizing the cost of resource procurement, resource provisioning among different edge clouds for mobile users should be considered carefully. Third, different types of tasks in an application have their own unique requirement and property. Some types of tasks can only be well handled by allocating more computing resources than other types. We need to carefully consider the amount of resources allocated to different types of task. Fourth, the geographical locations of edge clouds are varied, and mobile users may be dispersed and come into and leave system for service dynamically, making it hard to collect all users’ information in real time. In this case, centralized optimization strategies are uneasy to put into practice.

In this paper, we present a decentralized strategy based on an evolutionary game theory to address the above challenges. The evolutionary game theory is a promising solution for analyzing the dynamic behaviors of users under bounded-rationality, which has advantages over traditional game theoretic approaches. Traditional game theoretic approaches to model the interaction between players assume the complete rationality by maximizing their own payoffs. In addition, the solution of one traditional game cannot reflect the dynamics of players in the process of strategy adaption. Compared with classical game theory, evolutionary game theory considers a population instead of individual players. All players in the evolutionary game competition are divided into several populations. Players in the same population have the stationary proportion to adopt the same strategy under the assumption of limited rationality. By jointly considering resource allocation for different types of tasks and task offloading between different clouds, our decentralized strategy can obtain the evolutionary equilibrium, not requiring full knowledge of MEC system information. In our strategy, the cost of resource consumption in the MEC system is also minimized from the perspective of a mobile application provider. The major contributions of this research can be summarized as follows.

- We jointly formulate the problem of task offloading and resource allocation for mobile users in the MEC cloud platform as an evolutionary game, by taking account both resource procurement cost and users’ QoS. The populations of mobile users are modeled as players in the evolutionary game. The competition among players is to share the limited amount of resources in heterogeneous edge clouds and central clouds. By utilizing the process of replicator dynamics [8], we model the strategy adaption among different players.
- We design a decentralized strategy based on the evolutionary game theory to solve the problem of task offloading and resource allocation, following constraints of resource limitation in edge clouds and quality of experience (QoE) requirements of different tasks. Our strategy can ensure stability and can achieve an evolutionary equilibrium. We prove the stability and fairness of our strategy by mathematical analysis. The convergence rate is also analyzed in our work.
- We develop a discrete-event simulator based on OMNeT++ and conduct several trace-driven experiments. We compare the performance of our proposed strategy with other alternative methods and show that our method can not only satisfy the QoE requirement for players among different populations, but can also minimize the resource procurement cost.

The remainder of this paper is organized as follows. Section 2 reviews related works. Section 3 presents our system model and problem formulation. In Section 4, we present the evolutionary game formulation for task offloading problem. In Section 5, we design a decentralized strategy based on the evolutionary game theory. The convergence and stability analysis of our strategy is also presented in Section 5. In Section 6, we conduct several illustrative studies to evaluate the performance of our strategy. Finally, we conclude this paper and discuss possible future work in Section 7.

## 2. Related Work

In recently years, mobile applications, such as VR and AR, which can bring immersive experience to users by utilizing mobile crowd sensing [9], have attracted great attention from academia and industry. These mobile applications are changing different aspects of the world, such as online education, online game, and so on. With the commercialization and popularization of 5G network [10], mobile applications will have a higher quality network environment in the future. However, there are still several challenges for these applications to provide high QoS with flexible mobility. One of the vital challenges is that the computation capacity in mobile handsets will not grow effectively under the limitation of battery life [11]. Also, the technical advancement of Central Processing Units (CPUs) generally no longer follows the law of Moore [12]. Insufficient computing resources in a local mobile terminal will become a more and more challenging for above applications.

MEC opens another technical way to augment the computation capacity of mobile handsets. Previous researches such as [13,14] have done lots of works on the general task offloading and resource allocation, without distinguishing different types of applications. Cho et al. [15] and Chen et al. [16] focused on the task offloading for concrete applications, but these works have not considered that tasks in one application have different types, such as foreground and background rendering [11,17] for face recognition. These work also assumed that offloaded tasks share resources in a MEC cloud platform without distinction. Different types of tasks in the same application may have different resource requirements under delay constraint. It is not proper to allocate the same resources to perform different types of tasks. Compared with previous researches, our work mainly focuses on the optimization of one interactive application, considering task differences in the same application. To satisfy the demands of different types of tasks, we dynamically allocate appropriate amount of resources from the MEC cloud platform to mobile users.

Several teams proposed some optimizations in the MEC platform on the side of edge cloud, central cloud, or mobile users. Chen et al [14] and Ma et al. [18] proposed task offloading methods between one edge cloud and a central cloud. Plachy et al. [19] and Zhang et al. [20] proposed a task offloading strategy for multi-users among multiple edge clouds in the MEC. These works ignored the positive effect of the central cloud on edge clouds. Zhang et al. [21] proposed an auction-based service provider selection, utilizing both multiple edge clouds among multi-users. Compared with Zhang et al., Samimi et al. [22] proposed one similar task offloading strategy in cloud computing without considering the edge. But, the shortage of these work is that the interaction between users in one mobile application may happen among many individuals rather than just only two persons. Instant information exchange between edge clouds and central clouds is required for mobile interaction. Compared with these work, our work considers the cooperation of multiple edge clouds and a central cloud in the real environment and proposes an integrated strategy by considering multiple edge clouds to cooperate with a central cloud.

In terms of resource allocation and task offloading in a MEC system, different approaches have been proposed recently. Hou et al. [23] and Xu et al. [24] proposed strategies based on Markov Decision Process. But the state transition matrix in their proposed strategy sometimes could not be obtained precisely by historical statistic. Wang et al. [25] proposed a centralized strategy based on s-t graph cut to solve the maximum flow problem. This strategy should collect the whole system information to get the optimization results, which is not applicable in the distributed MEC system. Urgaonkar et al. [26] proposed a strategy based on Lyapunov theory, which focused on the optimization in the long-time term. However, it is not a practical solution because in each time slot, the QoE of task offloading may not be ensured. Aryal et al. [27] adopted the centralized genetic algorithm to address the NP-hard problem about heterogeneous resource allocation in a MEC system. Kuang et al. [28] proposed an agent-based framework to solve the problem of task offloading by one centralized heuristic strategy. Different from the above researches, a decentralized method has been exploited for the optimization of a MEC system. Gu et al. [29] proposed a decentralized task offloading strategy based on matching theory without considering the central cloud in the MEC cloud platform. Zhang et al. [30] proposed one coalition-game-based method to optimize the problem of task offloading. Players in this game should cooperate together to get the optimal strategy. However, mobile users in the real world have limited rationality. S. Jošilo et al. [31] and Xu et al. [14] proposed two similar game-based strategies for task offloading to achieve Nash equilibrium among mobile users. Players in these two game theoretic approaches aimed to maximize their own payoffs by considering other players’ behaviors. But, the shortage of their work is that even when one player changes his/her strategy, all other players should consider whether to change their strategies. It means that the Nash equilibrium [32] is unable to withstand minor disturbances. Different from previous work, based on the evolutionary game theory, our proposed method only need minimum information exchange from the MEC cloud platform, which fits greatly in a decentralized manner. In addition, our strategy can obtain the evolutionary equilibrium during the competition in the game, which can resist minor disturbances among players.

To sum up, our work differs from previous works in the following aspects: first, although there are some existing studies about the optimization for task offloading in the MEC cloud platform, these studies focused on task offloading without considering dynamic resource allocation between different types of tasks. Instead, we optimize the problem of task offloading by jointly considering appropriate resource allocation to each type of task in our strategy. Second, previous works concentrated on the task offloading optimization independently among one edge cloud and a central cloud or among multiple edge clouds. Instead, our work proposes an integrated solution by considering multiple edge clouds to cooperate with a central cloud. Third, compared with other approaches, our proposed method based on the evolutionary game theory only need minimum information from a MEC cloud platform, which fits greatly in a decentralized manner. Our algorithm can be proved by theoretical analysis, to be asymptotically stable and achieve to an evolutionary equilibrium.

## 3. Modeling and Formulation

We consider a typical MEC paradigm with multiple edge clouds, as shown in Figure 1. The paradigm, includes Mobile Users, Edge Cloud, a Central Cloud, and networks connecting them together. The focus of our study is to provide an optimized solution to offload tasks to appropriate Edge Clouds with appropriate VM assignment in the MEC platform. We will then introduce the service utility model and cost model in details in this section.

### 3.1. System Model

**Users and Tasks:** Mobile users in different geographical locations may offload their diverse types of tasks to edge clouds or central clouds on demand. Let U denote the set of all mobile users, I={1,2,3,...I} be the set of region, and $U_{i}$ be set of users in the *i*th region. Let J be the set of all offloaded task type, J={1,2,3...,J}. Let ${\hat{W}}_{j}$ be the average number of CPU cycles required to complete a type-*j* task, similarly as literature [33] does. ${\hat{L}}_{j}$ is denoted as the size of input and generated data in the type-*j* task. Let $U_{i}^{j}$ denote the set of users in the *i*th region, who need to perform the type-*j* task, and $u_{i}^{j}$ be the number of users in the set $U_{i}^{j}$.

**Edge Cloud:** Edge clouds in the MEC cloud platform are spread over different regions. Their service capacity are limited by their coverage area and resource. We denote the set of edge clouds as E={1,2,3...,E}. Let $C_{e}$ and $B_{e}$ be the capacity of computation and bandwidth for the *e*th edge cloud, respectively. In general, the MEC cloud platform fulfills an offloaded task via a set of virtual machines (VMs) or execution containers (e.g., Docker). To simplify our study without losing generality, we only consist using VMs. Let Q be the set of VM configuration types, Q={1,2,3...Q}. In the type-*q* VM configuration, the computation capacity is set as $A_{q}$ and the bandwidth capacity is set as $Z_{q}$. The cost of resource procurement in different edge clouds are also different, and we set VM rental cost of different types in different edge clouds as $V_{e}=\left\{v_{e}^{1},v_{e}^{2},...,v_{e}^{Q}\right\}$.

**Central Cloud:** Central clouds in the MEC cloud platform are also involved in task offloading for mobile users. Let K={1,2,3,...,K} be the set of central clouds in the MEC cloud platform. Task offloading to a central cloud also incurs a cost of resource consumption similar to edge clouds. Let $V_{k}=\left\{v_{k}^{1},v_{k}^{2},...,...v_{k}^{Q}\right\}$ be different types of VM rental cost in the *k*th central cloud. An interactive task need the information of other users as input, and the central cloud can collect and synchronize user status data with edge clouds. Hence, the central cloud undertakes a vital role in the interaction between mobile users.

### 3.2. Service Utility Model

The delay for completing an offloaded task is a vital factor that greatly affects users’ QoS. The delay is consisted of four parts: (1) The round-trip delay, which is incurred between users and their selected clouds. This delay can vary greatly due to diverse locations of users. We denote $d_{e}^{i}\left(r\right)$ as the round-trip delay between the *i*th region and *e*th edge cloud, and $d_{k}^{i}\left(r\right)$ as the round-trip delay between the *i*th region and *k*th central cloud. (2) The processing delay, which is incurred by task execution in the VM with type-*q* configuration. Let $d_{i}^{j}\left(p,q\right)$ be the processing delay for a task belonging to $U_{i}^{j}$ offloaded to a VM with type-*q* configuration. (3) The transmission delay, which is incurred by data transmission between users and their selected clouds. The input data and generated data need to be transferred between client and server side, in which incurs the transmission delay. Let $d_{i}^{j}\left(l,q\right)$ be the transmission delay for the task offloaded to a VM with type-*q* configuration. (4) The status synchronization delay. Considering user interaction, mobile users should synchronous status information when offloading tasks to edge clouds. Let $d_{o}$ be the sync delay.

To sum up, when a task belonging to $U_{i}^{j}$ is offloaded to a VM with type-*q* configuration in the *e*th edge cloud, the total delay to finish this task is as below:

(1) $$\begin{array}{c} d_{i}^{j}\left(e,q\right)=d_{o}+d_{i}^{j}\left(p,q\right)+d_{e}^{i}\left(r\right)+d_{i}^{j}\left(l,q\right) \end{array}$$

Similarly, when this task is offloaded to a VM of type-*q* configuration in the *k*th central cloud, the total delay is as below:

(2) $$\begin{array}{c} d_{i}^{j}\left(k,q\right)=d_{i}^{j}\left(p,q\right)+d_{i}^{j}\left(l,q\right)+d_{k}^{i}\left(r\right) \end{array}$$

According to the work [17], the task processing delay is not only related to allocated computation resource for the task, but also the load on the serving edge cloud or central cloud. When a task belonging to $U_{i}^{j}$ is offloaded to the *e*th edge cloud, the processing delay can be expressed as follows:

(3) $$\begin{array}{c} d_{i}^{j}\left(p,q\right)=\frac{r}{h^{A_{q}-{\hat{W}}_{j}}}d_{p} \end{array}$$

where $d_{p}$ is the task processing latency when the selected cloud is fully loaded, *h* represents the relationship between task computation demand and VM configuration, and *r* denote the current load in the selected cloud.

The transmission delay is related to the assignment of VM configuration, which can be computed as below:

(4) $$\begin{array}{c} d_{i}^{j}\left(l,q\right)=\frac{{\hat{L}}_{j}}{Z_{q}} \end{array}$$

### 3.3. Cost Model

From the perspective of mobile-service providers, the cost of completing an offloaded task varies greatly. The reasons are as follows: First, the unit cost of resource procurement in different edge clouds and central clouds are diverse. Second, different types of tasks are not assigned to the same type of VM configuration. The total costs of maintaining the mobile service for all users are consisted of two parts: VM rental cost in edge clouds and in central clouds. The total operational costs incurred in the *e*th edge cloud are as follows:

(5) $$\begin{array}{c} cost_{e}=\sum_{i=1}^{I}\sum_{j=1}^{J}\sum_{q=1}^{Q}n_{i}^{j}\left(e,q\right)v_{e}^{q}u_{i}^{j} \end{array}$$

where $n_{i}^{j}\left(e,q\right)$ indicates the radio of users belonging to $U_{i}^{j}$ that are routed to the VM with type-*q* configuration in the *e*th edge cloud.

Similarly, the total operational costs incurred in the *k*th central cloud are described as follows:

(6) $$\begin{array}{c} cost_{k}=\sum_{i=1}^{I}\sum_{j=1}^{J}\sum_{q=1}^{Q}n_{i}^{j}\left(k,q\right)v_{k}^{q}u_{i}^{j} \end{array}$$

where $n_{i}^{j}\left(k,q\right)$ indicates the radio of users in the set $U_{i}^{j}$ and all these users have been routed to the VM with type-*q* configuration in the *k*th central cloud.

Therefore, the total operational costs for the mobile service provider are as follows:

(7) $$\begin{array}{c} cost_{a}=\sum_{k=1}^{K}cost_{k}+\sum_{e=1}^{E}cost_{e} \end{array}$$

Without considering cost saving and resource limitation, the optimal strategy for offloaded tasks is obvious: each nearby edge cloud is selected by mobile users to assign the highest-configuration VM to serve tasks. However, the resource constraint in each edge cloud may limit the number of tasks to get response timely from the same edge cloud. An edge cloud may become overloaded when over-demanded tasks are offloaded to it, resulting in high task-processing delay. In addition, assigning all types of tasks to the same high configuration VM is not necessary and over-provisioning. Actually, an appropriate type configured VM is sufficient to ensure the delay requirement of a task. In our work, when offloading tasks between different clouds, we also consider assigning appropriate VM configuration to each type of task.

### 3.4. Joint Optimization Problem

**Figure 2:**
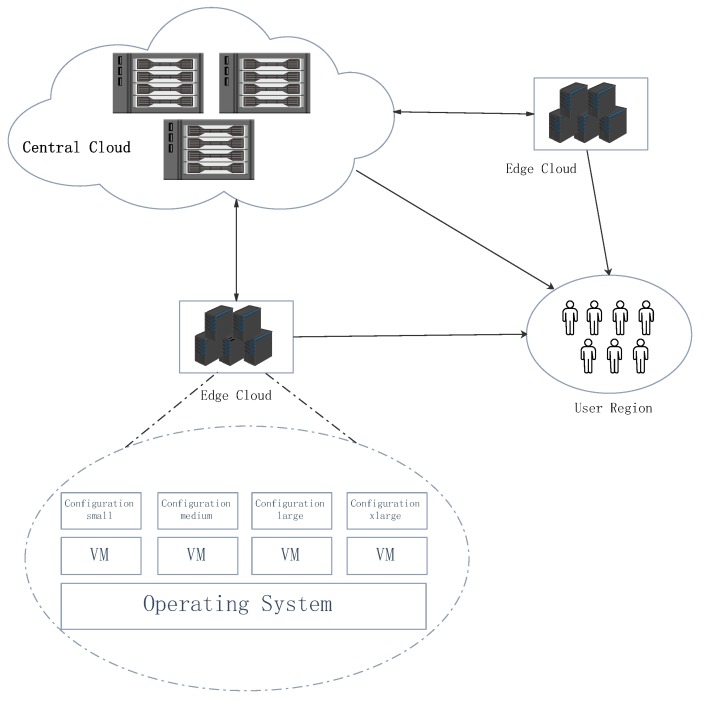
Task offloading and resource allocation in the MEC cloud platform.

Figure 2 depicts the problem of task offloading to different clouds and VM configuration assignment for different types of task, while jointly considering the delay constraint and resource procurement cost. The joint optimization problem is formulated as below:

(8) $$\begin{array}{cc} P1.\min & \;\;\;\sum_{k=1}^{K}cost_{k}+\sum_{e=1}^{E}cost_{e} \end{array}$$

subject to:

(9) $$\begin{array}{c} \sum_{k=1}^{K}\sum_{q=1}^{Q}n_{i}^{j}\left(k,q\right)+\sum_{e=1}^{E}\sum_{q=1}^{Q}n_{i}^{j}\left(e,q\right)=1 \end{array}$$

(10) $$\begin{array}{c} 0\leq n_{i}^{j}\left(e,q\right)\leq 1,\forall i\in I,\forall j\in J,\forall e\in E,\forall q\in Q \end{array}$$

(11) $$\begin{array}{c} 0\leq n_{i}^{j}\left(k,q\right)\leq 1,\forall i\in I,\forall j\in J,\forall k\in K,\forall q\in Q \end{array}$$

(12) $$\begin{array}{c} d_{i}^{j}\left(e,q\right)\leq \xi ,d_{i}^{j}\left(k,q\right)\leq \xi \end{array}$$

(13) $$\begin{array}{c} \sum_{i=1}^{I}\sum_{j=1}^{J}\sum_{q=1}^{Q}n_{i}^{j}\left(e,q\right)u_{i}^{j}A_{q}\leq C_{e},\forall e\in E \end{array}$$

(14) $$\begin{array}{c} \sum_{i=1}^{I}\sum_{j=1}^{J}\sum_{q=1}^{Q}n_{i}^{j}\left(e,q\right)u_{i}^{j}Z_{q}\leq B_{e},\forall e\in E \end{array}$$

where constraint (9) indicates that, each user belonging to $U_{i}^{j}$ should offload his/her task to an edge cloud or a central cloud, and VM with one configuration must be assigned to undertake the task process. The constraint (10) is similar with the constraint (11), which describes that the radio of users should be positive, no matter which clouds they choose to offload. The constraints (12) guarantees the delay requirement of tasks offloaded into edge clouds and central clouds. ξ is denoted as the maximum tolerant delay of tasks. To simplify the problem complexity, we only consider the same maximum delay tolerance for all types of tasks. The constraints (13) and (14) specify that the total amount of resources that users require from each edge cloud should not exceed the capacity of each edge cloud, including the computation resource and bandwidth resource.

To derive the problem **P1** with the above constraints, we can design a centralized strategy by applying the dual decomposition [34] to transform the above constrained problem to an equivalent, unconstrained Lagrangian. However, the dual decomposition converges slowly owing to its huge variable space, and should collect the information of all users. Furthermore, due to the heterogeneity and dynamics join and leave behaviors of mobile users, this centralized optimization strategy based on all user information is inefficient and impractical. In the next section, we develop a decentralized approach based on evolutionary game theory to solve the problem of dynamic task offloading and resource provisioning.

## 4. Evolutionary Game Theoretic Strategy

In this section, we first give the evolutionary game formulation for the problem of task offloading and VM configuration assignment in the MEC cloud platform. Due to the limitation of computation resource and bandwidth resource in each edge cloud, mobile users in different regions should compete for resources in clouds. Based on the replicator dynamics, we design an evolutionary stable strategy (ESS) to adapt strategies among users. The stability and equilibrium of our strategy is analyzed.

### 4.1. Evolutionary Game Formulation

A normal game includes three factors: the player set, the strategy set, and the payoff function of every player when choosing a strategy. In the context of an evolutionary game, the population is utilized to represent the group of players with the same properties. We introduce the formulation of the evolutionary game as below:

**Set of Players:** Mobile users in diverse geographical locations are denoted as players in the game.

**Set of Strategies:** An individual should offload his/her task to an edge cloud or a central cloud with an assignment of VM configuration. The combination set of cloud selection and VM configuration assignment is defined as the strategy space for players. If a central cloud is chosen, we treat it as a special edge cloud with a great amount of resources. Accordingly, let S be the action set for all players. The number of strategies that each player can choose is (E+K)Q in total. Hence, we can simply represent a strategy *s* as {s=(e,q)|e∈E,q∈Q} or {s=(k,q)|k∈K,q∈Q}.

**Populations:** Players are grouped into different populations by task types and geographical locations. We denote the set of population as $\left\{U_{1}^{1},U_{1}^{2},U_{2}^{2},...,U_{I}^{J}\right\}$. Players in each population are all located in the same geographical region. All players in the same population have the same type of task for offloading. We denote $u_{i}^{j}\left(s\right)$ as the number of players selecting strategy *s* for the population $U_{i}^{j}$. The population share of adopting strategy *s* in the population $U_{i}^{j}$ is given as:

(15) $$\begin{array}{c} n_{i}^{j}\left(s\right)=\frac{u_{i}^{j}\left(s\right)}{u_{i}^{j}}, \end{array}$$

**Payoff:** The utility of a player in this game is mainly decided by task processing time and resource procurement cost. The cloud selection and VM configuration assignment would both affect the completion process of offloaded tasks.

When a task is offloaded into an edge cloud, the workload of the edge cloud is given:

(16) $$\begin{array}{c} r_{e}=\frac{\sum_{i=1}^{I}\sum_{j=1}^{J}\sum_{q=1}^{Q}n_{i}^{j}\left(e,q\right)\left(u_{i}^{j}A_{q}\right)}{C_{e}} \end{array}$$

Then, we can give the QoE gain of users, which can reflect the delay of completing a task belonging to $U_{i}^{j}\left(e,q\right)$ as below. In this QoE expression, players can obtain a higher value, when the delay of completing a task is smaller.

(17) $$\begin{array}{c} QoE_{i}^{j}\left(e,q\right)=\xi -\left(d_{o}+\frac{r_{e}}{h^{A_{q}-{\hat{W}}_{j}}}d_{e}+d_{e}^{i}\left(r\right)+d_{i}^{j}\left(l,q\right)\right) \end{array}$$

In the utility function of a player, a player is set to receive a penalty for the VM rental cost, by considering the problem of resource over-provisioning. Furthermore, to show the importance of scarce resources in edge clouds, the penalty function shown below is defined to follow the idea that a thing is valued more if it is rare.

(18) $$\begin{array}{c} Pen_{i}^{j}\left(e,q\right)=r_{e}v_{e}^{q} \end{array}$$

The expected utility of a player offloading his/her task to an edge cloud is denoted as the QoE gain minus the cost penalty as below:

(19) $$\begin{array}{c} \pi_{i}^{j}\left(e,q\right)=\gamma_{1}QoE_{i}^{j}\left(e,q\right)-\gamma_{2}Pen_{i}^{j}\left(e,q\right), \end{array}$$

where $\gamma_{1}$ and $\gamma_{2}$ are the weight between QoE gain of users and penalty for costs.

When a task is offloaded to a central cloud, the workload of the central cloud is assumed to be unchanged, due to the large scale of resource in central clouds. Therefore, the workload in each central cloud is set to be close to a constant δ as below:

(20) $$\begin{array}{c} r_{k}=\delta \end{array}$$

The QoE gain of a task offloaded into the *k*th central cloud is similar to that into the edge cloud:

(21) $$\begin{array}{c} QoE_{i}^{j}\left(k,q\right)=\xi -\left(\frac{r_{k}}{h^{A_{q}-{\hat{W}}_{j}}}d_{k}+d_{k}^{i}\left(r\right)+d_{i}^{j}\left(l,q\right)\right) \end{array}$$

The penalty of a task offloaded into a central cloud is:    

(22) $$\begin{array}{c} Pen_{i}^{j}\left(k,q\right)=v_{k}^{q} \end{array}$$

The utility of a player offloading his/her task to a central cloud is:

(23) $$\begin{array}{c} \pi_{i}^{j}\left(k,q\right)=\gamma_{1}QoE_{i}^{j}\left(k,q\right)-\gamma_{2}Pen_{i}^{j}\left(k,q\right) \end{array}$$

### 4.2. Evolutionary Stable Strategy

In a traditional game theory, all players can achieve a stable state where no player can further obtain extra benefit by unilaterally changing its strategy. Such a state is called Nash equilibrium. In the next equation, we call the game Γ as the game of cloud selection and VM configuration assignment, U as the player set, S as the strategy set of all players, and Π as the set of payoff function. Let $s_{-u}=\left\{s_{1},...,s_{u-1},s_{u+1},...s_{U}\right\}$ be a strategy profile of all players except player *u*, and $\pi (s_{-u},s_{u})$ is set to be the payoff function of player *u* when this player selects the strategy $s_{u}$ while others select $s_{-u}$. Then, we give the precise definition of Nash equilibrium as below.

**Definition** **1.** A Nash equilibrium (NE) of the resource allocation game Γ=<U,S,Π> is a strategy profile $S^{*}=\left\{s_{1}^{*},s_{2}^{*},...,s_{U}^{*}\right\}$ such that $\pi \left(s_{-u}^{*},s_{u}\right)\leq \pi \left(s_{-u}^{*},s_{u}^{*}\right),\forall s_{u}\in S$.

The Nash equilibrium has a property of self-reinforcement that each player has no motivation to deviate from this equilibrium. The general solution to obtain the Nash equilibrium is on the assumption of complete rationality among all players. However, with a small perturbation, all players may change their strategies to reach another Nash equilibrium. In an evolutionary game theory, an equilibrium strategy is adopted among players with bounded rationality, which can resist small disturbances. This equilibrium strategy is called ESS and is defined as below.

**Definition** **2.** A strategy profile $S^{*}$$=\{s_{1}^{*},s_{2}^{*},...,s_{U}^{*}\}$ is an ESS if and only if, $\forall s_{u}\notin S^{*}$ and $s_{-u}\neq s_{-u}^{*}$:

*1.*  $\pi \left(s_{-u}^{*},s_{u}\right)\leq \pi \left(s_{-u}^{*},s_{u}^{*}\right)$.
*2.*  if $\pi \left(s_{-u}^{*},s_{u}\right)=\pi \left(s_{-u}^{*},s_{u}^{*}\right)$, $\pi \left(s_{-u},s_{u}\right)<\pi \left(s_{-u},s_{u}^{*}\right)$.

Compared with Nash equilibrium, the condition (1) of Definition 2 ensures that ESS is a Nash equilibrium (NE). The condition (2) of Definition 2 ensures the stability of the game process. During the process of strategy evolution, players using mutation strategy will decrease until all players in the population asymptotically approach to the ESS.

In our problem, mobile users adapt their strategies among a finite set of strategies to get a better payoff. In each time, each mobile user can have his/her own strategy set and the information of average payoff in the same population. Each mobile user can repeatedly evolve his/her strategy over time for the cloud selection and VM configuration assignment. After sufficient repetitive stages, all mobile users’ strategy profile approaches to an ESS. The process of this strategy replication can be modeled by replicator dynamics, which is described in the next section.

### 4.3. Replicator Dynamics

In a dynamic evolutionary game, an individual in a population would adapt his/her strategy by comparing his/her payoff with average payoff in the same population. The individual would adapt to another strategy that if his/her payoff is lower than the average payoff. Then, the population share that adopts different strategies will evolve over time until each player in the same population achieves the same payoff. The process of strategy selection for different populations can be modeled as ordinary differential equations, called replicator dynamics. It is given as below:

(24) $$\begin{array}{c} {\dot{n}}_{i}^{j}\left(s\right)=\sigma n_{i}^{j}\left(s\right)\left(\pi_{i}^{j}\left(s\right)-\bar{\pi }\left(i,j\right)\right) \end{array}$$

where σ is used to control the convergence speed of strategy adaption for players in the same population. $\bar{\pi }\left(i,j\right)$ is the average payoff in the population of $U_{i}^{j}$, which can be computed from $\bar{\pi }\left(i,j\right)=\sum_{s=1}^{S}n_{i}^{j}\left(s\right)\pi_{i}^{j}\left(s\right)$.

Based on the replicator dynamics of strategy selection in the population $U_{i}^{j}$, the number of mobile users that choose the strategy *s* has a positive growth trend in the population if their payoff is above the average payoff in the same population. Through setting ${\dot{n}}_{i}^{j}\left(s\right)=0$, we can get the fixed point of the replicator dynamics, in which the population state will not change and no player is willing to change its strategy since all players in the same population have the same payoff. The expression in (24) can be transformed as below:

(25) $$\begin{array}{c} {\dot{n}}_{i}^{j}\left(s\right)=\sigma n_{i}^{j}\left(s\right)\left(\pi_{i}^{j}\left(s\right)-\sum_{s=1}^{S}n_{i}^{j}\left(s\right)\pi_{i}^{j}\left(s\right)\right)=0 \end{array}$$

According to Definition 1, we can prove that all solutions to the above algebraic equations belong to the Nash equilibrium. In some literature such as Ref. [35], they are also called evolutionary equilibrium for the evolutionary game. The number of solutions to (25) can be proved to be at least one above. We show this in the following remark.

**Remark** **1.** *The evolutionary game of cloud selection and VM configuration assignment has at least one evolutionary equilibrium.*

**Proof.**  When all mobile users in the same population choose the same cloud, $n_{i}^{j}\left(x\right)\in \left\{0,1\right\},\forall x\in S$. And, $n_{i}^{j}\left(x\right)$ can be proved to satisfy Expression (25). In this situation, we name this type of solution to Equation (25) as bounded evolutionary equilibrium.

When $n_{i}^{j}\left(x\right)\in \left(0,1\right),\exists x\in S$, we can simplify Expression (25) as $\pi_{i}^{j}\left(s\right)-\sum_{s=1}^{S}n_{i}^{j}\left(s\right)\pi_{i}^{j}\left(s\right)=0$. Due to $\sum_{s=1}^{S}n_{i}^{j}\left(s\right)=1$, we can obtain that $\sum_{s^{*}=1}^{S}n_{i}^{j}\left(s^{*}\right)\left(\pi_{i}^{j}\left(s\right)-\pi_{i}^{j}\left(s^{*}\right)\right)=0,\forall s\in S$. If there exists one solution that can satisfy $\pi_{i}^{j}\left(s\right)-\pi_{i}^{j}\left(s^{*}\right)=0,\forall s\in S$, we can deduce that this solution is one of evolutionary equilibrium, which is equivalent to the solution of Equation (26). We call this solution as interior evolutionary equilibrium.

(26) $$\left\{\begin{array}{c} \pi_{i}^{j}\left(s\right)-\pi_{i}^{j}\left(s^{*}\right)=0\\ \sum_{s=1}^{S}n_{i}^{j}\left(s\right)=1 \end{array}\;\forall s\in S\right.$$

We can prove that there exists one solution to (26). $\pi_{i}^{j}\left(s\right)=\gamma_{1}QoE_{i}^{j}\left(s\right)-\gamma_{2}Pen_{i}^{j}\left(s\right)$ is related to the load of an edge cloud. When the number of mobile users is large, $\pi_{i}^{j}\left(s\right)$ would decrease a lot if mobile users offload huge amount of tasks to the same edge cloud beyond its computation capacity. Mobile users can get better payoff if they choose another edge cloud. $n_{i}^{j}\left(x\right)$ can be balanced during the strategy adaption in the evolutionary game. In summary, we can find at least one evolutionary equilibrium for the evolutionary game of cloud selection and VM configuration assignment. □

Through analyzing the algebraic Equation (25), we can obtain two types of evolutionary equilibrium, namely, bounded evolutionary equilibrium and interior evolutionary equilibrium. According to Definition 2, the ESS can be obtained by solving the asymptotically stable equilibrium of the replicator dynamics. The bounded evolutionary equilibrium, which does not satisfy condition (2) of the ESS definition, cannot resist the invasion of small perturbation to the equilibrium state. The interior evolutionary equilibrium has the property of stability in the evolutionary game. We show this conclusion in the following theorem.

**Theorem** **1.** *For the cloud selection and VM configuration assignment, the interior evolutionary equilibrium in our game is asymptotically stable.*

**Proof.**  We denote an interior evolutionary equilibrium by $\hat{n}\left(i,j\right)=\left\{{\hat{n}}_{i}^{j}\left(1\right),{\hat{n}}_{i}^{j}\left(2\right),...{\hat{n}}_{i}^{j}\left(S\right)\right\}$. Define the tracking error function $e_{i}^{j}\left(s\right)={\hat{n}}_{i}^{j}\left(s\right)-n_{i}^{j}\left(s\right)$, and the Lyapunov function of the above system is defined as $V_{i}^{j}\left(s\right)=\frac{{\left(e_{i}^{j}\left(s\right)\right)}^{2}}{2}$. We can get $V_{i}^{j}\left(s\right)\geq 0$ all the time.

The time derivative of $V_{i}^{j}\left(s\right)$ is presented as below:

(27) $$\begin{array}{cc} {\bar{V}}_{i}^{j}\left(s\right) & =\frac{\partial ({\left(e_{i}^{j}\left(s\right)\right)}^{2}/2)}{\partial t}=e_{i}^{j}\left(s\right)\frac{\partial (e_{i}^{j}\left(s\right))}{\partial t}=-e_{i}^{j}\left(s\right)\frac{\partial (n_{i}^{j}\left(s\right))}{\partial t}\\ & =\;-\sigma \left({\hat{n}}_{i}^{j}\left(s\right)-n_{i}^{j}\left(s\right)\right)n_{i}^{j}\left(s\right)\left(\pi_{i}^{j}\left(s\right)-\bar{\pi }\left(i,j\right)\right)\;\\ & =\;-\sigma \left({\hat{n}}_{i}^{j}\left(s\right)-n_{i}^{j}\left(s\right)\right)n_{i}^{j}\left(s\right)\left(\pi_{i}^{j}\left(s\right)-\sum_{s=1}^{S}n_{i}^{j}\left(s\right)\pi_{i}^{j}\left(s\right)\right)\; \end{array}$$

When the utility function $\pi_{i}^{j}\left(s\right)>\bar{\pi }\left(i,j\right)$, the population of players $U_{i}^{j}$ that adopts the strategy *s* is in a number of growth stage. Due to $n_{i}^{j}\left(s\right)\neq 0$, $n_{i}^{j}\left(s\right)$ will increase according to expressions (24) and (25), which means that $n_{i}^{j}\left(s\right)<{\hat{n}}_{i}^{j}\left(s\right)$. So, we can prove that the time derivative of $V_{i}^{j}\left(s\right)$ is negative. Based on the Lyapunov stability theory [36], the dynamic strategy selection of mobile users is asymptotically stable. Therefore, the replicator dynamics of mobile users choosing different strategies will converge to the interior evolutionary equilibrium, which belongs to an ESS. □

### 4.4. Delay in Replicator Dynamics

In the previous description of replicator dynamics, each mobile user decides on cloud selection and VM configuration assignment based on average payoff in the same population. In fact, each mobile user may not get the average payoff instantly. The delay may be incurred by mobile network transmission latency. Under this situation, players in the evolutionary game could only utilize the historical information to make the optimal decisions. In this case, we assume that the delay of information updating in the evolutionary game is set to be τ. The delayed replicator dynamics can be modified as below:

(28) $$\begin{array}{c} {\dot{n}}_{i,j}^{s}\left(t\right)=\sigma n_{i,j}^{s}\left(t-\tau \right)\left(\pi_{i,j}^{s}\left(t-\tau \right)-{\bar{\pi }}_{i,j}\left(t-\tau \right)\right), \end{array}$$

where a mobile user makes decisions at the time slot *t*, based on the historical information at the time slot t−τ. To investigate the impact of delay in the replicator dynamics, we also analyze and prove the convergence of delayed replicator dynamics by using the Lyapunov method [37] as below.

**Theorem** **2.** *The convergence of delayed replicator dynamics can be guaranteed in the evolutionary game of task offloading by dynamic cloud selection and VM configuration assignment.*

**Proof.**  Similar with the proof processing of theorem (1), we set one Lyapunov function $V_{i,j}^{s}\left(t\right)=\frac{{\left(e_{i,j}^{s}\left(t\right)\right)}^{2}}{2}$, where $e_{i,j}^{s}\left(t\right)={\hat{n}}_{i,j}^{s}\left(t\right)-n_{i,j}^{s}\left(t\right)$.

The time derivative of $V_{i,j}^{s}\left(t\right)$ is presented as below:

(29) $$\begin{array}{cc} {\bar{V}}_{i,j}^{s}\left(t\right) & =\frac{\partial ({\left(e_{i,j}^{s}\left(t\right)\right)}^{2}/2)}{\partial t}=-e_{i,j}^{s}\left(t\right)\frac{\partial (n_{i,j}^{s}\left(t\right))}{\partial t}\\ & =\;-\;\sigma \left({\hat{n}}_{i}^{j}\left(s\right)\;-\;n_{i}^{j}\left(s\right)\right)n_{i,j}^{s}\left(t\;-\;\tau \right)\left(\pi_{i,j}^{s}\left(t-\tau \right)-{\bar{\pi }}_{i,j}\left(t-\tau \right)\right)\; \end{array}$$

When the payoff is less than the average payoff meaning that $\pi_{i,j}^{s}\left(t-\tau \right)-{\bar{\pi }}_{i,j}\left(t-\tau \right)<0$ in the time slot t−τ, the number of players adopting the strategy *s* will decrease at the time slot *t*. So, the time derivative of $V_{i,j}^{s}\left(t\right)$ is negative. Based on the Lyapunov method, we can conclude that the delayed replicator dynamics would finally converge to the interior evolutionary equilibrium. □

We also explore the impact of delay on the stability of delayed replicator dynamics by conducting simulations in Section 6, to confirm the robustness of our theory.

## 5. Iterative Algorithm Design and Analysis

In this section, we show the implementation of an iterative algorithm based on the replicator dynamics to help task offloading for mobile users by dynamic cloud selection and flexible VM configuration assignment. The performance of our algorithm is also analyzed in this section.

### 5.1. Online Algorithm Implement

In our iterative algorithm implement, most mobile users select their optimal strategies at the initial stage. A few mobile users randomly choose their strategies. The MEC cloud platform would calculate the average payoff in each population and send the information of the load in the MEC cloud platform and average payoff in each population back to mobile users. Then, mobile users can decision in their own terminals about task offloading by utilizing this information to get a higher payoff. After many iterations, mobile users would dynamically adapt their strategies iteratively until their payoff are close to the average payoff in the same population.

At each iteration, the strategy adaption processing can be described as one iterative replicator:

(30) $$\begin{array}{c} n_{i,j}^{s}\left(t\right)\;=\;n_{i,j}^{s}\left(t\;-\;\tau \right)\;+\;\sigma n_{i,j}^{s}\left(t\;-\;\tau \right)\left(\pi_{i,j}^{s}\left(t-\tau \right)-{\bar{\pi }}_{i,j}\left(t-\tau \right)\right), \end{array}$$

where σ is used to control the iterative step size. The iteration’s terminating criterion is set as follows:

(31) $$\begin{array}{c} \left|\pi_{i,j}^{s}\left(t\right)-{\bar{\pi }}_{i,j}\left(t\right)\right|<\epsilon , \end{array}$$

where ϵ is a small positive parameter, and τ is the delay of information update during the strategy adaption in our game. The detail of the iterative algorithm is summarized in the Algorithm 1, which is named as IASVA for short.

**Algorithm 1** Iterative Algorithm on Cloud Selection and VM Configuration Assignment (IASVA)
1:**Initialization:** For most mobile users, task offloading strategies are selected to maximize their own utility; Several mobile users select strategies by random.2: 3:**while** the condition (31) is not satisfied **do**4: 5:     Each mobile user offloads his/her task to an assigned VM in the selected edge cloud. Then, the edge cloud collect the payoff information of each mobile user $\pi_{i,j}^{s}\left(t\right)$ to the central cloud.6: 7:     The central cloud compute the average payoff ${\bar{\pi }}_{(}i,j)$, and send back to each edge cloud.8: 9:     Mobile users change their strategies with probability σ, when their payoff is less than the average payoff in the same population.10: 11:     Update t=t+τ.12: 13:
**end while**



### 5.2. Performance Analysis

From the perspective of the mobile service provider, the objective is to cost-effectively allocate limited resources to mobile users, while considering fairness and utility gain among mobile users. We analyze the performance of our strategy on the side of utility gain, time efficiency, and fairness.

We will show that our iterative strategy can obtain the Pareto-optimal utility gain when our strategy has only one ESS. For different strategies of task offloading, the Pareto-optimal strategy profile [38] has the following feature:

**Definition** **3.** *A strategy profile $X=\{x_{1},x_{2},...x_{U}\}$ is Pareto-optimal if there does not exist any strategy profile $X^{\prime }=\left\{x_{1}^{\prime },x_{2}^{\prime },...x_{U}^{\prime }\right\}$ such that $\pi_{u}\left(x_{u}^{\prime }\right)\geq \pi_{u}\left(x_{u}\right),\forall u\in U$, with at least one strict inequality.*

We will analyze and prove this property by using the method of contradiction as below.

**Theorem** **3.** *Our iterative strategy of task offloading for mobile users in the MEC platform is Pareto-optimal when converging to the ESS that bring the maximal social welfare among all the ESSes, and is local Pareto-optimal when converging to another ESS.*

**Proof.**  When our evolutionary game has only one ESS, this strategy is Pareto-optimal. We show this by contradiction. Assume this sole evolutionary strategy *X* is not Pareto-optimal. Then, there exists a Pareto-optimal allocation $X^{\prime }$ such that $\pi_{u}\left(x_{u}^{\prime }\right)\geq \pi_{u}\left(x_{u}\right),\forall u\in U$. According to the definition of ESS, the small disturbance of strategy adaption would not change the dominant strategy of users. So, $\pi_{u}\left(x_{u}^{\prime }\right)\geq \pi_{u}\left(x_{u}\right),\forall u\in U$ cannot occur in the evolutionary game. Thus, the sole ESS in our evolutionary game is Pareto-optimal.

If a system has multiple evolutionary stable strategies, the strategy that can generate the greatest utility, has the property of Pareto-optimal. We can prove that this strategy reach the strong Nash equilibrium, which is both Pareto optimum and Nash equilibrium of the stage game according to Ref. [39]. Other evolutionary stable strategies have the property of local Pareto-optimal, which achieves the Pareto-optimal within the range of the ESS. □

Considering the property of time efficiency, our proposed iterative algorithm IASVA can be deployed efficiently in the real distributed environment. At each iteration, the time complexity of our distributed algorithm focuses on step 3 and step 4, which can be completed in a small constant time. The iteration number of our algorithm is related to the parameter σ and initial state. It can roughly be estimated as $\left|\frac{n_{i,j}^{s}\left(0\right)-{\hat{n}}_{i,j}^{s}}{\sigma }\right|$, where $n_{i,j}^{s}\left(0\right)$ is the initial stage of mobile users selecting different strategies and ${\hat{n}}_{i,j}^{s}$ is the stable evolutionary equilibrium of mobile users. To evaluate the performance of our algorithm, we analyze the convergence speed of our iterative strategy in detail as below.

First, we denote an interior evolutionary equilibrium by ${\hat{n}}_{i,j}^{s}=\left\{{\hat{n}}_{i,j}^{1},{\hat{n}}_{i,j}^{2},...{\hat{n}}_{i,j}^{s}\right\}$. We construct a gap function $G_{i,j}^{s}=\left(n_{i,j}^{s}-{\hat{n}}_{i,j}^{s}\right)u_{i}^{j},{\hat{n}}_{i,j}^{s}\neq 0$ between the number of current mobile users and the stable fixed point that decide the strategy *s*.

Let $E_{i,j}^{s}\left(t\right)$ denote the number of mobile users in the population $U_{i}^{j}$ to change their strategies at time t, and we have

(32) $$E_{i,j}^{s}\left(t\right)=\delta n_{i,j}^{s}\left(t-\tau \right)u_{i}^{j},G_{i,j}^{s}\left(t-\tau \right)>0$$

where δ is denoted as the mutation probability.

In the case of $G_{i,j}^{s}\left(t-\tau \right)>0$, at time *t*, we can get that $G_{i,j}^{s}\left(t\right)=X_{i,j}^{s}\left(t\right)-{\hat{n}}_{i,j}^{s}u_{i}^{j}$, where $X_{i,j}^{s}\left(t\right)$ is the current number of user in the population $U_{i}^{j}$ adopting strategy *s*. $X_{i,j}^{s}\left(t\right)$ satisfies that $X_{i,j}^{s}\left(t\right)=X_{i,j}^{s}\left(t-\tau \right)-E_{i,j}^{s}\left(t\right)$.

To evaluate the convergence speed of our iterative algorithm, we define one expression as below:

(33) $$\beta =|\frac{G_{i,j}^{s}\left(t\right)}{G_{i,j}^{s}\left(t-\tau \right)}\left|=\right|\frac{X_{i,j}^{s}\left(t-\tau \right)-\delta n_{i,j}^{s}\left(t-\tau \right)u_{i}^{j}-{\hat{n}}_{i,j}^{s}u_{i}^{j}}{X_{i,j}^{s}\left(t-\tau \right)-{\hat{n}}_{i,j}^{s}u_{i}^{j}}|$$

According to the rate of convergence [40], 0<β<1 can be utilized to prove that our algorithm is linearly convergent to the interior evolutionary equilibrium. So, our iterative algorithm would be linearly convergent to the interior evolutionary equilibrium if we select one mutation probability small enough that satisfies the above inequality.

## 6. Illustrative Studies

To evaluate the performance of our iterative algorithm, we develop a discrete-event simulator based on the OMNeT++ [41] to simulate the environment of MEC cloud platform and behaviors of mobile users. We conduct a set of experiments on the customized simulator to show the advantages of our strategy over other alternative strategies.

### 6.1. Experiment Setting

In the simulator, the MEC cloud platform has a central cloud and multiple edge clouds. Similar to Ref. [42], the MEC platform is deployed in a network environment with hexagonal cellular structures. In the simulated MEC platform, we assume that there are three base stations (eNodeB), which are distributed evenly in a square area with dimensions of 3 km × 3 km. We partition the area into three regions of the same size. Each region is well covered by one eNodeB. An edge cloud is deployed in each region close to one eNodeB. According to the LTE-Advanced network [43], we set the channel band to be 20 MHz. The transmission power of each eNodeB is set to be 50 dBm, so every eNodeB can cover each region of mobile users even considering the pass loss factor. The background noise is set to be −100 dBm [33] for each mobile user. Each Mobile user in one region can share up to 100 Million bits per second (Mbps) for the down-link channel bandwidth, and up to 50 Mbps for the up-link channel bandwidth.

Round-trip delay between a user in one region and an edge cloud is a function of the distance between them. We set a round-trip delay between a mobile user and an edge cloud to be in the range of [10 ms, 20 ms] at the same region, to be in the range of [100 ms, 200 ms] at different regions. The round-trip delay between mobile users and the central cloud is set to be in the range of [200 ms, 500 ms]. The computation resources in each edge cloud can be described as the number of CPU cycle [14,33]. We set the CPU computation capacity of each edge cloud to be one from the set {$2\times 10^{2}$ GHz, $2.5\times 10^{2}$ GHz, $3\times 10^{2}$ GHz}, and the CPU computation capacity of the central cloud is set to be $3\times 10^{4}$ GHz [44]. We assume that each cloud has three types of VM configurations for handling offloaded tasks. The computing capacity for each VM configuration is assumed to be one from the set {1 GHz, 2 GHz, 3 GHz}. The bandwidth configuration of VM is assumed to be from the set {2 Mbps, 4 Mbps, 8 Mbps}. The unit VM rental cost of different configurations in each edge cloud and central cloud is assumed to in the range of (0,1) dollar per hour.

Mobile users in our experiments may generate different types of tasks dynamically. All tasks are grouped into three types, which is set to consume the number of CPU cycle in the set {10 Megacycles, 20 Megacycles, 30 Megacycles}. Each task can be completed satisfactorily if a right corresponding configured VM is assigned for it. The generated data from these three types of tasks are consumed to be in the set {200 KB, 400 KB, 800 KB} [33].

### 6.2. Methodology

We also simulate some other alternative strategies to verify the superior of our proposed strategy in terms of QoE performance and cost saving. One optimal centralized method with delay constraint is introduced for comparison, which is also shown in the formulation of problem **P1**. The optimal centralized method considers solving the constrained problem P1 based on the dual decomposition, which is named as Central in our comparison. Two greedy methods, namely Cost-Only and Delay-Only, are also used to evaluate the performance boundary of our strategy. The Cost-Only method is aimed at saving the overall operational costs for the mobile service provider where his or her tasks are offloaded to an edge cloud with lowest operational costs. The Delay-Only method considers improving the QoE of a mobile user with no upper-bound budget where tasks are offloaded to a cloud that can process tasks the fastest. To enhance the evaluation of our strategy, we also introduce one decentralized method [33] based on Nash equilibrium, called Nash-based, which treats tasks equally without considering dynamically allocating computation resources to different types.

### 6.3. Experiment Results and Analysis

#### 6.3.1. Performance Comparison

We evaluate the performance by comparing the operational cost and task completion time obtained from our strategy with those from other alternatives vs. the number of offloaded task.

**Figure 3:**
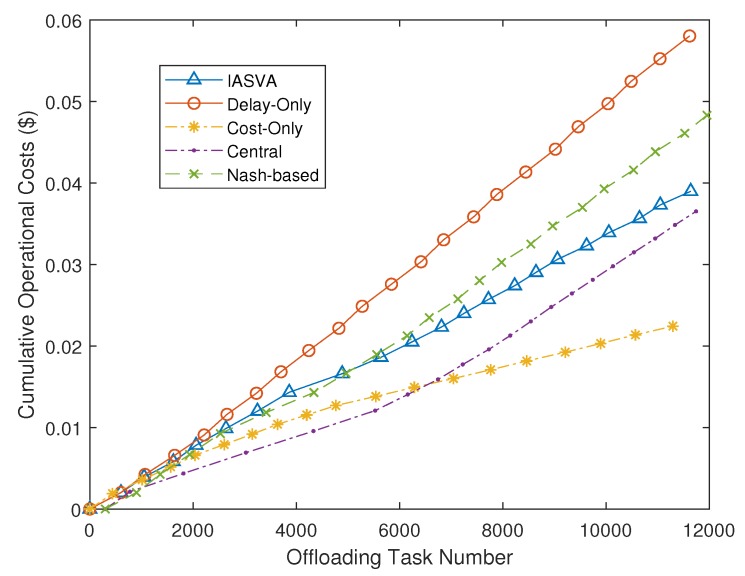
Cumulative operational costs of five strategies.

Figure 3 shows the cumulative operational costs of five strategies. We observe that the cumulative costs incurred by our strategy IASVA are lower than Cost-Only and Central strategy. The operational costs incurred by the Nash-based strategy is higher than that incurred by our strategy. This is because our strategy can dynamically allocate resources to different types of tasks under the delay constraint. The Nash-based strategy sometimes would lead to the problem of resource over-provisioning for tasks. The gap between the overall cost of our strategy and that of the optimal solution is also presented in Figure 3. The optimal strategy is a centralized strategy, which need collect the whole information of all mobile users. Although the optimal strategy can save more operational costs, our decentralized strategy can be more suitable to the practical environment.

**Figure 4:**
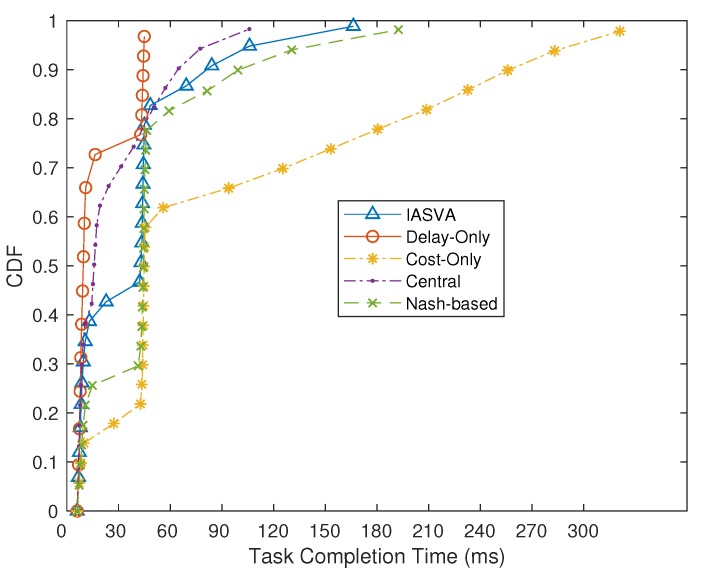
Task-completion time of four strategies.

Figure 4 shows the cumulative distribution function (CDF) of task-completion time of different strategies. From this figure we can observe that, in most cases, the task-completion time incurred by our strategy is close to that incurred by the Central strategy. The Nash-based strategy would achieve higher task completion time than that incurred by our strategy. This is because our strategy would allocate higher configuration VM to tasks, when task processing time affect the user’s QoS. The central strategy can obviously obtain the lower task processing time than ours. However, in the practical environment, collecting all information of users in the central strategy would incur additional delay, which would aggressively affect user’s QoS.

#### 6.3.2. Fairness Evaluation

**Figure 5:**
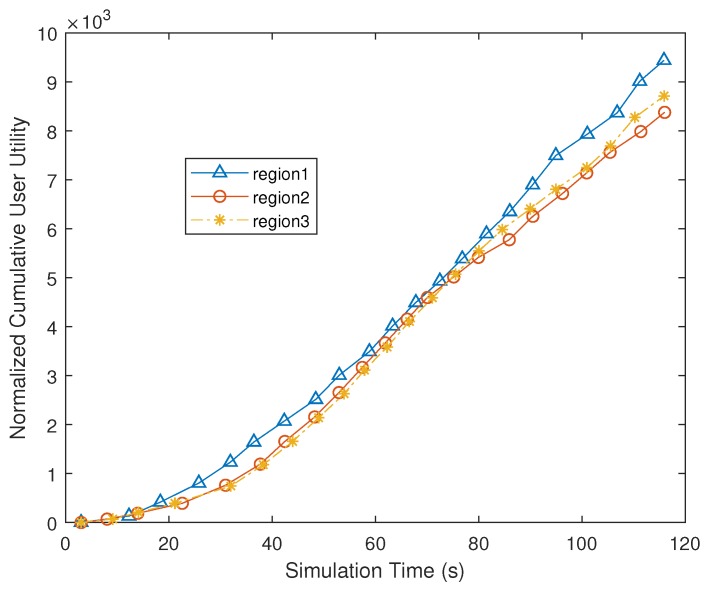
Cumulative user utility in different regions.

To show the fairness, we also compare the user utility among different regions. Figure 5 depicts the cumulative user utility in different regions when our strategy is used. We can observe that the cumulative utility obtained from these three regions are close to each other, clearly confirming that our decentralized strategy can maintain fairness among users of different regions.

#### 6.3.3. Stability of Our Proposed Strategy

**Figure 6:**
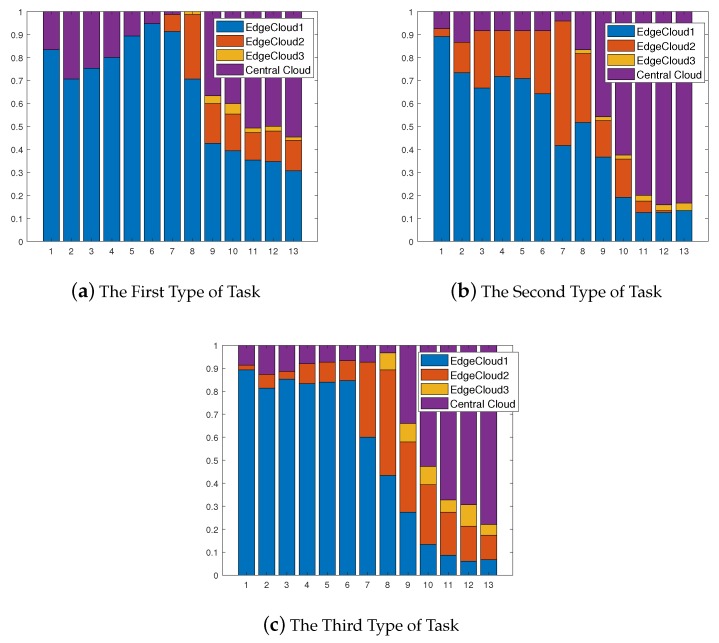
Dynamic Cloud Selection for Mobile Users in the Same Region.

Figure 6 shows the adaption of cloud selection radio among mobile users in the same region along with iterative number. Figure 6a–c presents the cloud selection radio among mobile users who offload three different types of tasks, separately. We can observe that the proportion of cloud selection between edge clouds and the central cloud gradually converge in the three sub-figure, respectively. The adaption of cloud selection among mobile users in the same region can show the stability of our strategy.

**Figure 7:**
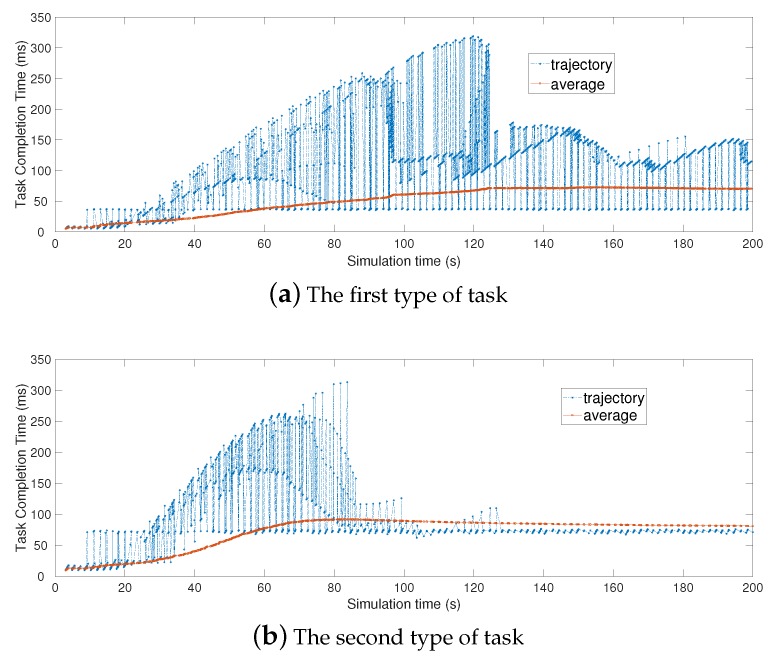

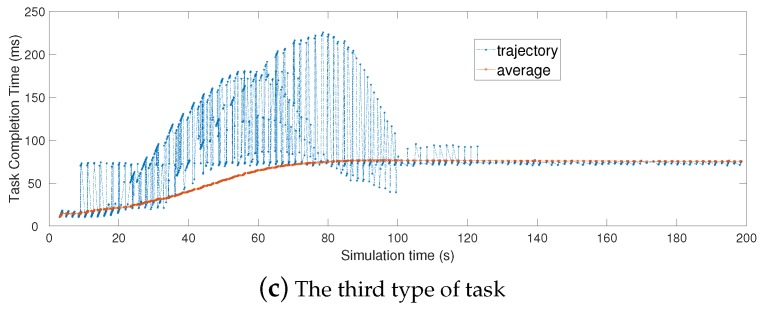
Different types of task completion time in the same region.

Figure 7 shows the trajectories of three types of task completion time in the same region. The change trend of average completion time for the same type of task in Figure 7 is also shown. In Figure 7a, mobile users, who all upload the first type of tasks, would eventually experience the stable task completion time, just as the same with Figure 7b,c.

To sum up, we can observe that the system converges to the asymptotically stable status after several simulation time.

#### 6.3.4. Adaptiveness of Our Proposed Strategy

**Figure 8:**
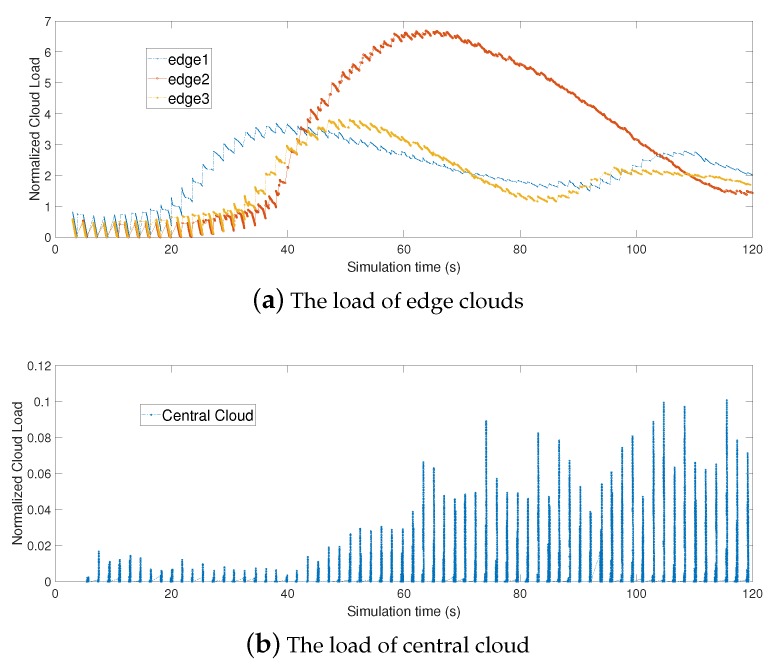
The normalized load of clouds along with simulation time.

**Figure 9:**
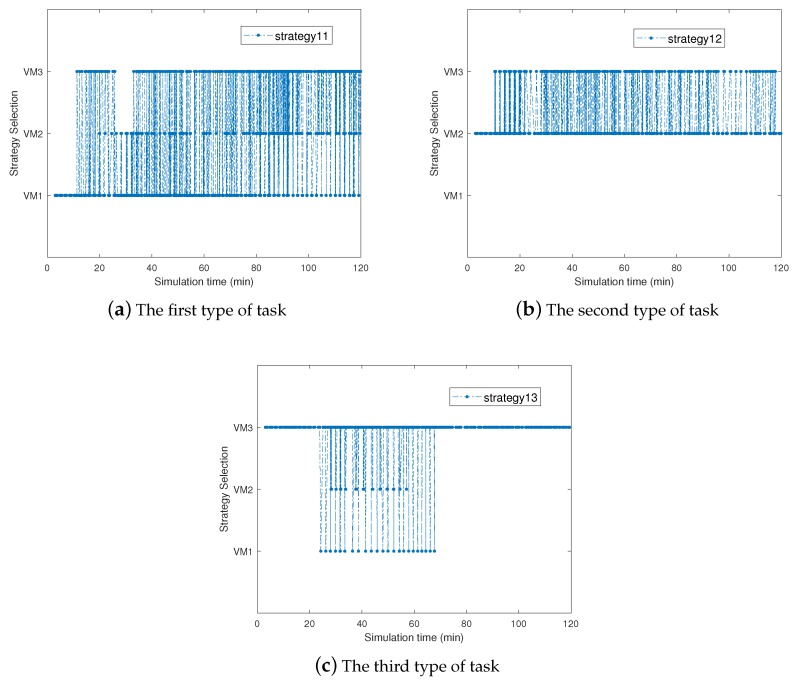
Dynamic VM Assignment for Different Types of Task in the Same Region.

To evaluate the adaptability of our algorithm to the dynamics of a practical environment, we conduct experiments to show the process of strategy evolution for relative sufficiently operation time: (1) the load in different edge clouds and the central cloud; (2) dynamic VM Assignments for different types of tasks in the same region. Figure 8 describes the change of load in the edge clouds and the central cloud. In our iterative strategy, three edge clouds finally get the same load. The load in edge clouds is related to the division between allocated computation resources and edge cloud’s processing capacity. Our iterative algorithm can dynamically allocate computation resources to different types of tasks. When the load in an edge cloud is high, mobile users would apply lower computation resources in this edge cloud to perform his/her offloading tasks or select other clouds for task offloading so that the load can be controlled. So, our strategy can adaptively decide the number of offloading tasks and resource allocation according to the computation capacity of edge clouds. Figure 9 shows the dynamic VM Assignments for different types of tasks in the same region. Based on the simulation results, our strategy can help a mobile user to adaptively decide VM assignment when the load of edge cloud is high. As a result, the loads of edge clouds decrease and the idle resources in the central cloud are utilized as well by the joint optimization of cloud selection and VM assignment.

#### 6.3.5. Impact of Delay in Information Exchange

**Figure 10:**
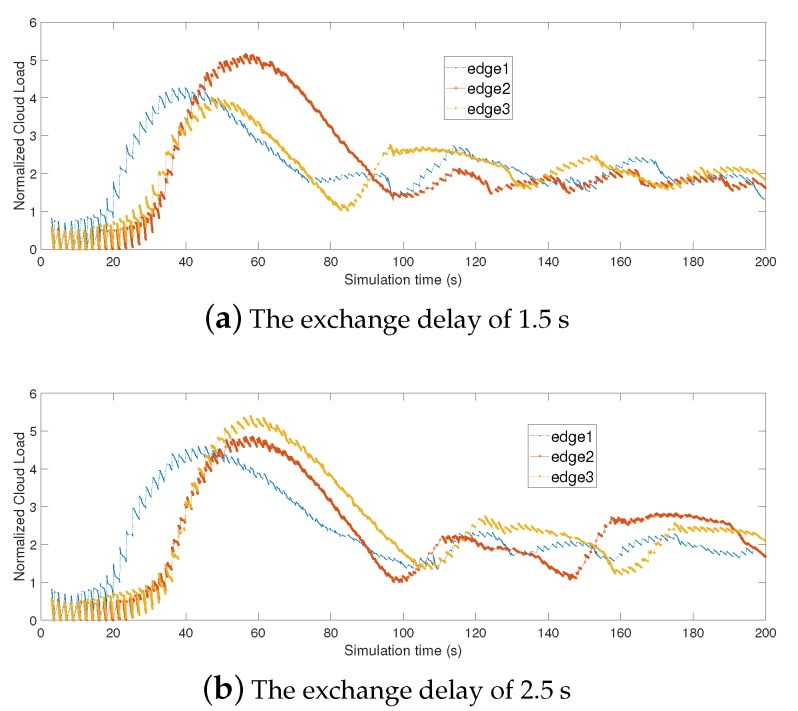

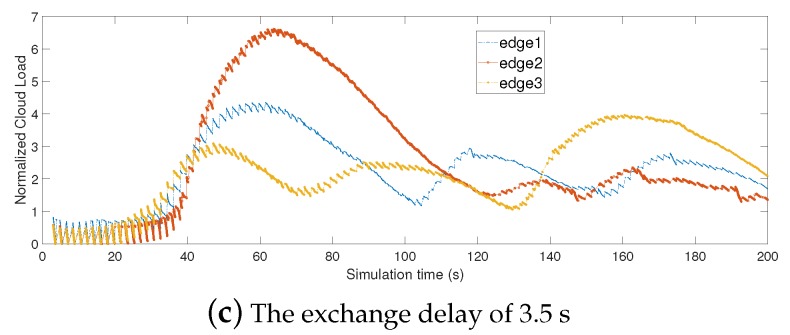
The convergence speed incurred by information exchange delay.

To evaluate the impact of information exchange delay, we utilize the load of edge cloud to show the convergence speed of our strategy. Figure 10 shows the normalized load of different edge clouds under different information exchange delay. It shows that the convergence speed of our strategy is related to the information exchange delays, at a lower convergent speed when the delay increases.

## 7. Conclusions

We investigated the problem of task offloading in the MEC cloud platform. To overcome the unsustainable power-consumption requirement and shortage of computation resources in mobile terminals, optimally offloading tasks to the MEC cloud platform effectively and efficiently is a promising approach to derive it. To meet QoS for users and extend battery life, we proposed a decentralized and iterative algorithm to help mobile users optimally offload their tasks in the MEC cloud platform based on evolutionary game theory. Our proposed iterative strategy can properly decide, for each mobile user, a proper edge cloud to serve with right VM configuration. Through a series theoretical analysis, we demonstrated that our strategy is asymptotically stable and can converge to an ESS in several iterations.

To validate the theoretical analysis and evaluate the effectiveness of the strategy, we conducted illustrative studies using a trace-driven simulations based on OMNet++ with a MEC cloud platform similar to the practical environment, and simulated dynamical behaviors of mobile users. From these illustrative studies, we confirmed that our strategy achieves closely to the minimal operational cost within a small difference, while ensuring the QoE of mobile users.

In our future work, we plan to consider the different preferences of mobile users at a live MEC cloud environment.
